# Co-Infection of Mosquitoes with Chikungunya and Dengue Viruses Reveals Modulation of the Replication of Both Viruses in Midguts and Salivary Glands of *Aedes aegypti* Mosquitoes

**DOI:** 10.3390/ijms18081708

**Published:** 2017-08-04

**Authors:** Alain Le Coupanec, Stéphane Tchankouo-Nguetcheu, Pascal Roux, Huot Khun, Michel Huerre, Ronald Morales-Vargas, Margot Enguehard, Dimitri Lavillette, Dorothée Missé, Valérie Choumet

**Affiliations:** 1 Unité de Génétique Moléculaire des Bunyavirus, Institut Pasteur, 75015 Paris, France; Alain.LeCoupanec@iaf.inrs.ca (A.L.C.); stephane.tchankouo@gmail.com (S.T.-N.); 2 Imagopole, Institut Pasteur, 75015 Paris, France; pascal.roux@pasteur.fr; 3 Unité de Recherche et d’Expertise Histotechnologie et Pathologie, Institut Pasteur, 75015 Paris, France; hkhun@pasteur.fr (H.K.); michel.huerre@curie.fr (M.H.); 4 Department of Medical Entomology, Faculty of Tropical Medicine, Mahidol University, Bangkok 73170, Thailand; ronald.mor@mahidol.ac.th; 5 Interspecies transmission of arboviruses and Therapeutics research Unit, Institut Pasteur of Shanghai, Shanghai Chinese Academy of Sciences, Shanghai 200031, China; margot.enguehard@etu.univ-lyon1.fr (M.E.); dlaville@ips.ac.cn (D.L.); 6 Maladies infectieuses et vecteurs: écologie, génétique, évolution et contrôle (MIVEGEC), IRD, 34394 Montpellier, France; dorothee.misse@ird.fr; 7 Unité Environnement et Risques Infectieux, Institut Pasteur, 75015 Paris, France

**Keywords:** *Aedes aegypti*, arbovirus, chikungunya, dengue, co-infection

## Abstract

Arthropod-borne virus (arbovirus) infections cause several emerging and resurgent infectious diseases in humans and animals. Chikungunya-affected areas often overlap with dengue-endemic areas. Concurrent dengue virus (DENV) and chikungunya virus (CHIKV) infections have been detected in travelers returning from regions of endemicity. CHIKV and DENV co-infected *Aedes albopictus* have also been collected in the vicinity of co-infected human cases, emphasizing the need to study co-infections in mosquitoes. We thus aimed to study the pathogen-pathogen interaction involved in these co-infections in DENV/CHIKV co-infected *Aedes aegypti* mosquitoes. In mono-infections, we detected CHIKV antigens as early as 4 days post-virus exposure in both the midgut (MG) and salivary gland (SG), whereas we detected DENV serotype 2 (DENV-2) antigens from day 5 post-virus exposure in MG and day 10 post-virus exposure in SG. Identical infection rates were observed for singly and co-infected mosquitoes, and facilitation of the replication of both viruses at various times post-viral exposure. We observed a higher replication for DENV-2 in SG of co-infected mosquitoes. We showed that mixed CHIKV and DENV infection facilitated viral replication in *Ae. aegypti*. The outcome of these mixed infections must be further studied to increase our understanding of pathogen-pathogen interactions in host cells.

## 1. Introduction

Arthropod-borne virus (arbovirus) infections cause several emerging and resurgent infectious diseases in humans and animals. Arboviruses are unusual in that they replicate in both arthropod and vertebrate hosts. Traditional means of controlling the spread of arbovirus infection include vaccination of susceptible vertebrates and mosquito control. However, these measures are often either unavailable or ineffective [1]. Further knowledge of virus/vector interactions is required to successfully implement the strategy of blocking the virus at the insect stage [2]. 

Three requirements must be fulfilled for an arthropod to serve as an efficient arbovirus vector. The arthropod must ingest a sufficient quantity of viremic blood to infect gut cells. After entering gut cells, sufficient viral replication must occur so that the virus can enter the hemocoel and infect other tissues such as salivary glands (SG) [3,4]. Finally, there must be sufficient multiplication at this latter site to ensure transmission within the saliva during the arthropod’s bite [5]. 

Amongst the various blood-feeding arthropods, *Ae. aegypti* is one of the most anthropophilic and cosmotropical of mosquito vectors. It has been implicated in several outbreaks of dengue, chikungunya, yellow fever, and other arboviruses. 

Chikungunya virus (CHIKV) is an emerging mosquito-borne pathogen. This virus is an alphavirus of the *Togaviridae* family; containing an 11.8 Kb single-stranded linear RNA genome of positive polarity enveloped by a 70 nm diameter capsule [6,7]. It has a major impact on human health, causing fever, headache, rash, nausea, vomiting, myalgia, and arthralgia. The virus is indigenous to tropical Africa, but there have been reports of widespread outbreaks in parts of South East Asia and several of its neighbouring islands in 2005–2007 and in Europe in 2007 [8]. CHIKV reached the Americas in 2013 and has since caused more than a million cases of infection [9]. Currently, there is no vaccine or antiviral treatment against CHIKV. CHIKV has a short extrinsic incubation period in *Ae. aegypti* and in *Ae. albopictus* mosquitoes and is present in the saliva as early as 2 days post-infection in this latter species [10].

Dengue virus (DENV) belongs to the *Flaviviridae* family and the Flavivirus genus. Its viral genome consists of a positive sense RNA of ~11  kb. Dengue fever is a globally important arboviral infection transmitted by mosquitoes of the *Aedes* genus (primarily *Aedes aegypti*, but also *Aedes albopictus*). Dengue infection causes a range of mild and severe clinical manifestations [11,12,13]. The infection is now endemic in more than 100 countries, particularly the South East Asia region, the Western Pacific region, and the Americas [14]. Severe manifestations such as dengue haemorrhagic fever and dengue shock syndrome, as well as other unusual manifestations, are increasingly being reported in previously unaffected regions. The mean extrinsic incubation period of dengue viruses is 8–12 days at 25 °C [15].

Although CHIKV-affected areas often overlap with DENV-endemic areas [16], simultaneous outbreaks are rare or undetected. However, northwest Gabon and Libreville were struck by a simultaneous CHIKV and DENV serotype 2 (DENV-2) outbreak in 2007, during which nine patients were co-infected by the two viruses [17,18]. Moreover, co-infections with chikungunya and dengue viruses were reported in India, Colombia and Guatemala [19,20,21]. Concurrent dengue and chikungunya virus infections have been detected in travelers returning from regions of endemicity [22,23,24]. These coinfections may be explained either by the consecutive bites of differentially infected mosquitoes or by bites of co-infected mosquitoes. *Aedes albopictus* co-infected with CHIKV and DENV have been collected in the vicinity of human cases emphasizing the need to study coinfections in mosquitoes [18]. Orally CHIKV and DENV co-infected *Aedes albopictus* from La Réunion Island were shown to deliver both viruses in their saliva [25].

In this work, we used reverse transcription polymerase chain reaction (RT-qPCR), immunofluorescence, and immunohistochemistry (IHC) to measure and compare the infection kinetics of CHIKV and DENV serotype 2 (DENV-2) in *Ae. aegypti* mosquitoes either infected singly or by both viruses. The results obtained show that each virus modified the infection kinetics of the other. We observed higher titres in the midguts (MG) and SG at several time points post-infection, potentially leading to a higher transmission efficiency. 

## 2. Results

### 2.1. Detection of Chikungunya Virus (CHIKV) and Dengue Virus Serotype 2 (DENV-2) Antigens by Immunofluorescence Assay (IFA) in Orally Infected Ae. aegypti Females

**Figure 1:**
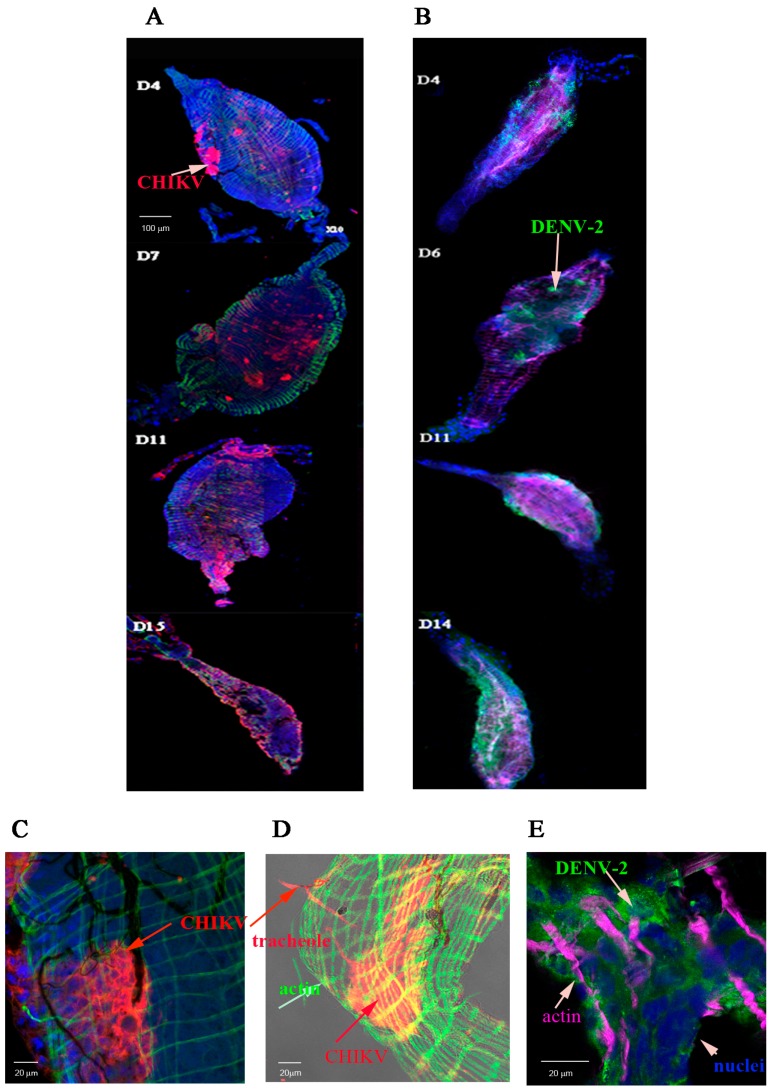
Distribution of viral antigens in midguts infected by chikungunya virus (CHIKV) and dengue virus serotype 2 (DENV-2). Midguts (MG) were collected at days 4, 7, 11, and 15 post-virus exposure for CHIKV and 4, 6, 11, and day 14 post-virus exposure for DENV-2 as indicated in panels (**A**,**B**) respectively. Viral antigens were labelled using anti-CHIKV 3E4 (panel (**A**) in red) or anti-DENV-2 4E11 (panel (**B**) in green) monoclonal antibodies. Nuclei were stained with DAPI and the actin network with Alexafluor 488 (panel (**A**) in green) or 633 (panel (**B**) in magenta) phalloidin. The distribution of CHIKV antigen in a CHIKV-infected midgut at day 3 post-virus exposure was shown in panels (**C**,**D**). Muscular tissue stained in green with Alexafluor 488 phalloidin (**C**,**D**); CHIKV antigens labelled in red with Cy3-labelled 3E4 anti-E protein (**C**,**D**) and nuclei in blue (DAPI) (**C**). (**D**) shows a surperimposition of a transmission light picture to an immunofluorescence picture. The distribution of DENV-2 antigens in the posterior part of the midgut at day 3 post-virus exposure was shown in panel **E**. The muscular tissue is shown in magenta (Alexafluora 633 phalloidin), viral antigens in green (4E11 monoclonal antibody) and nuclei in blue (DAPI).

We first investigated the distribution of CHIKV and DENV-2 particles in MG and SG of *Ae. aegypti* following infection with either CHIKV or DENV-2 on various days post-virus exposure (dpve). Infection foci for CHIKV and DENV-2 were clearly observable in the MG at 4 dpve. By days 11 to 15 post-virus exposure, the entire MG was labelled for both viruses, but the labelling was more diffuse (Figure 1A,B). Midguts were observed at a higher magnification. CHIKV antigen was visible in the cytoplasm of MG cells in the posterior part of the CHIKV-infected MG at day 3 post-virus exposure (Figure 1C) as well as in gut-associated tracheoles (Figure 1D). DENV-2 antigen foci were visible in cells of a DENV-2 infected MG at day 3 post-virus exposure (Figure 1E).

**Figure 2:**
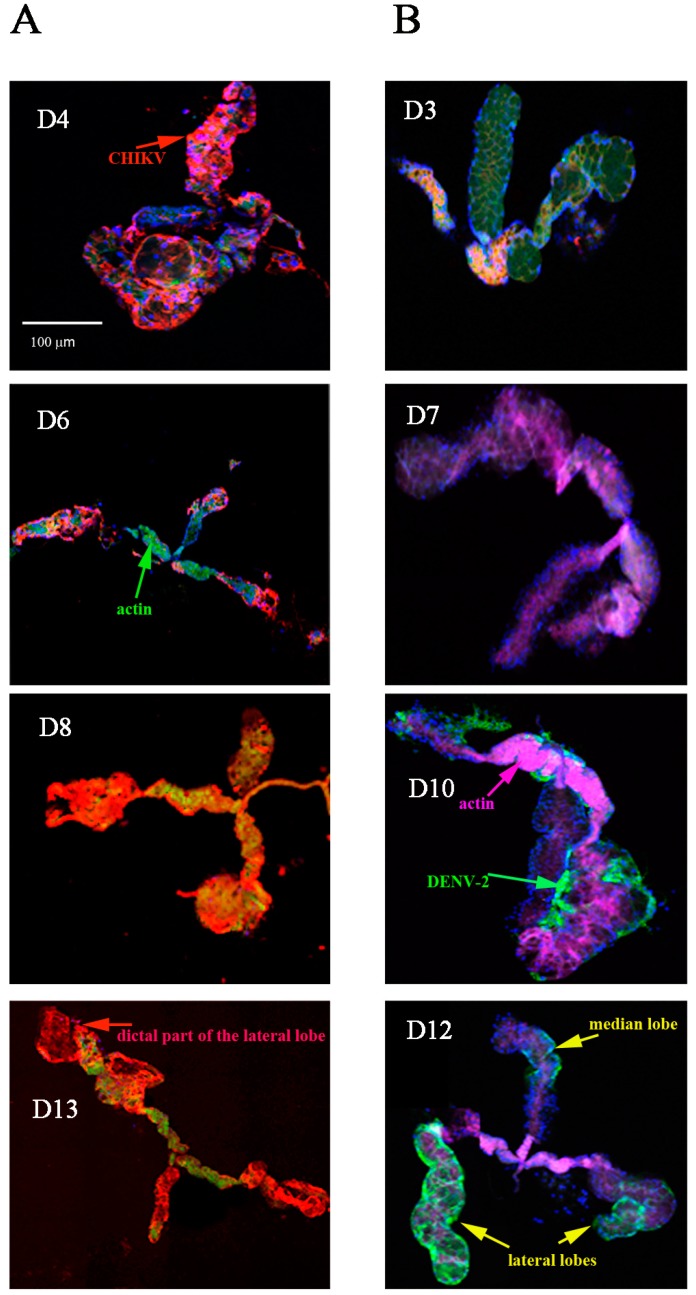

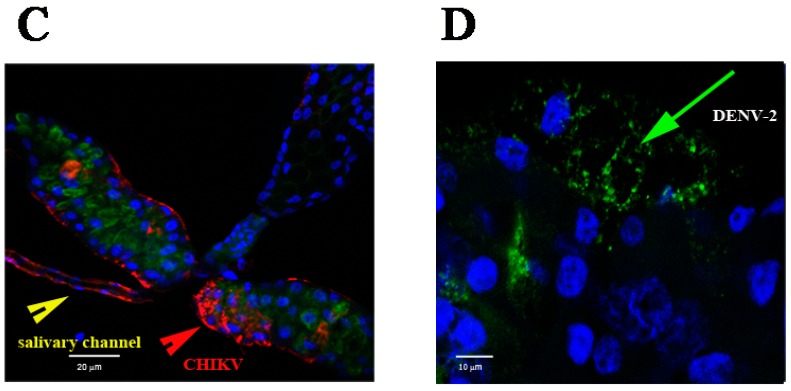
Distribution overtime of viral antigen in salivary glands infected by CHIKV and DENV-2. SG were collected at days 4, 6, 8, and 13 post-virus exposure for CHIKV and days 3, 7, 10, and 12 post-virus exposure for DENV-2 as indicated in panels A and B respectively. Viral antigens were labelled using anti-CHIKV 3E4 (panel (**A**) in red) or anti-DENV-2 4E11 (panel (**B**) in green) monoclonal antibodies. Nuclei were stained with DAPI and the actin network with Alexafluor 488 (panel (**A**)) or 633 (panel (**B**)) phalloidin; (**C**) CHIKV-infected salivary gland at day 11 post-virus exposure. CHIKV is labelled in red (Cy3-labelled 3E4 anti-E protein), the actin network in green (Alexafluor 488 phalloidin), and the nuclei in blue (DAPI). The salivary channel is shown by an arrow; (**D**) DENV-2 infected salivary gland at high magnification. The virus is labelled in green (4E11 anti-E protein monoclonal antibody) and nuclei in blue (DAPI).

CHIKV antigen was visible as early as 4 dpve in the SG, which increased slightly in area and intensity by day 8 post-virus exposure (Figure 2A). DENV-2 antigen was not visible in the SG until day 10 post-virus exposure (Figure 2B). The two viruses were found in the three lobes of each SG as well as in cells of the salivary channel for CHIKV (Figure 2C). We observed structural evidence of virus-associated pathology in the SG. The distal lateral lobes were distended, and some disrupted, from day 8 post-virus exposure for CHIKV and day 10 post-virus exposure for DENV-2, as previously shown for Sindbis virus [26]. These effects were more pronounced in CHIKV-infected cells than in those infected with DENV-2. We detected no morphological damage in the median lobes. Figure 2D shows distribution of DENV-2 antigen at a high magnification in the cytoplasm of adjacent cells of the proximal part of a SG lateral lobe at day 10 post-virus exposure.

**Figure 3:**
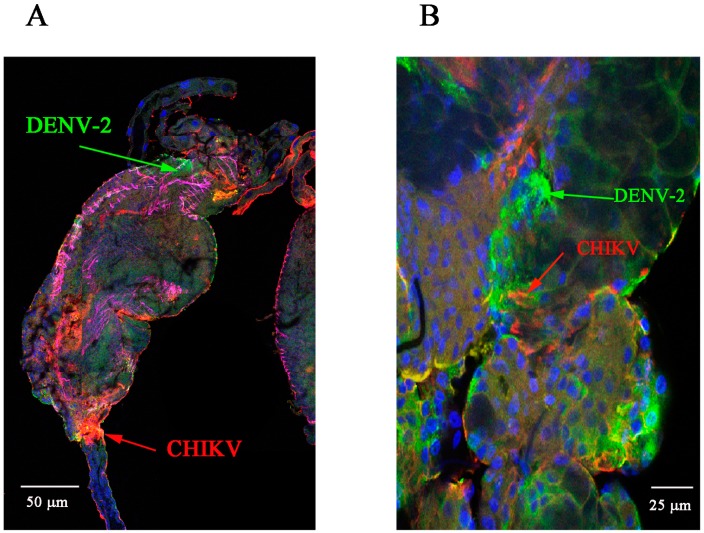

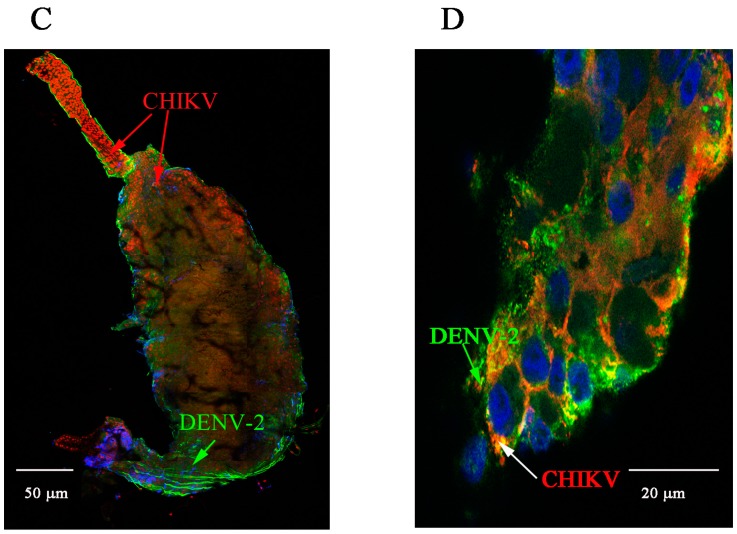
Detection of viral antigens in CHIKV/DENV-2 co-infected midguts and salivary glands at various days post-virus exposure. (**A**,**C**) Midgut at days 7 and 10 post-viral exposure respectively. CHIKV is labelled in red (Cy3-labelled 3E4 monoclonal antibody), DENV-2 in green (4E11 anti-E monoclonal antibody), nuclei in blue (DAPI), and the actin network in magenta (Alexafluor 633 phalloidin); (**B**,**D**) Salivary gland at days 10 and 13 post-viral exposure respectively. DENV-2 antigen is shown in green (4E11 anti-E monoclonal antibody), CHIKV antigen in red (Cy3-labelled 3E4 monoclonal antibody), and nuclei in blue (DAPI).

We then co-infected mosquitoes with DENV-2 and CHIKV and collected MG and SG at various times post-viral exposure. We detected both CHIKV and DENV-2 antigens in MG collected at 7 and 10 dpve (Figure 3A,C). We also detected both CHIKV and DENV-2 antigens in SG at 10 and 13 dpve (Figure 3C,D). Examination of the distribution of the two viruses in the distal part of the lateral lobe of SG collected at 10 and 13 days post-viral exposure showed double staining in many cells (Figure 3B,D).

### 2.2. Immunolocalisation of Virus in Midgut (MG) and Salivary Gland (SG) by Immunohistochemistry (IHC) Following Oral Exposure to DENV-2, CHIKV, or Both

We determined the spatial and temporal distribution of CHIKV and DENV-2 antigens in sections of paraffin-embeded mosquitoes after oral exposition to both viruses. For co-infection experiments, since double staining of the same section did not give interpretable results, we differentially stained various sections from the same co-infected mosquito using anti-CHIKV or anti-DENV-2 antibodies.

**Figure 4:**
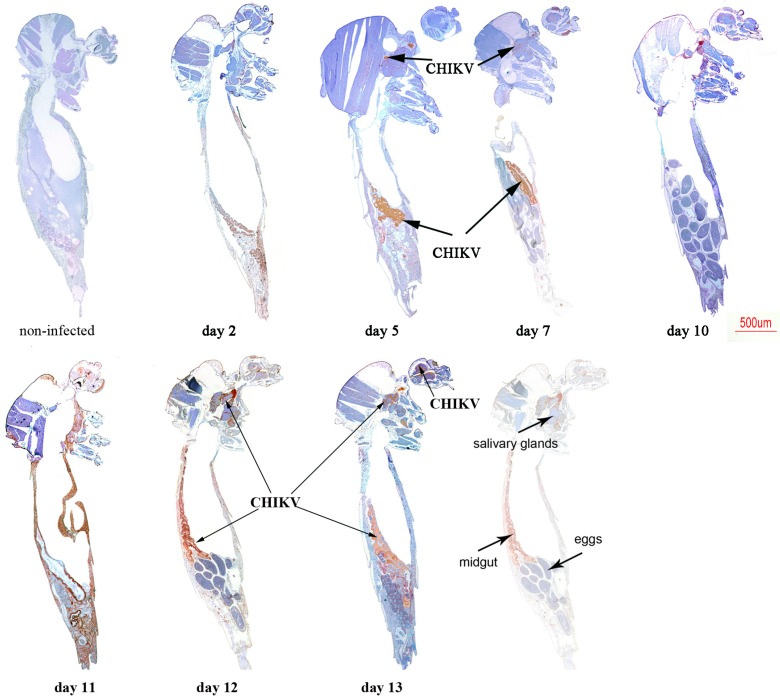
Immunohistochemical (IHC) staining of CHIKV in paraffin-embedded mosquitoes at various times post-virus exposure. CHIKV antigen stains brownish-red. A non-infected mosquito was used as control to determine non-specific staining using 3E4 anti-CHIKV monoclonal antibody.

We first analysed mono-infected mosquitoes. CHIKV antigens were detected at day 2 post-virus exposition in MG (Figure 4), while they started to be observed in SG at day 5 and increased overtime until day 13.

Heads were found infected at day 7 until the end of the experiment at day 13. CHIKV antigen was faintly detected in ovarian follicles at days 10 and 12, but interestingly eggs were not stained as shown at days 10 and 12.

**Figure 5:**
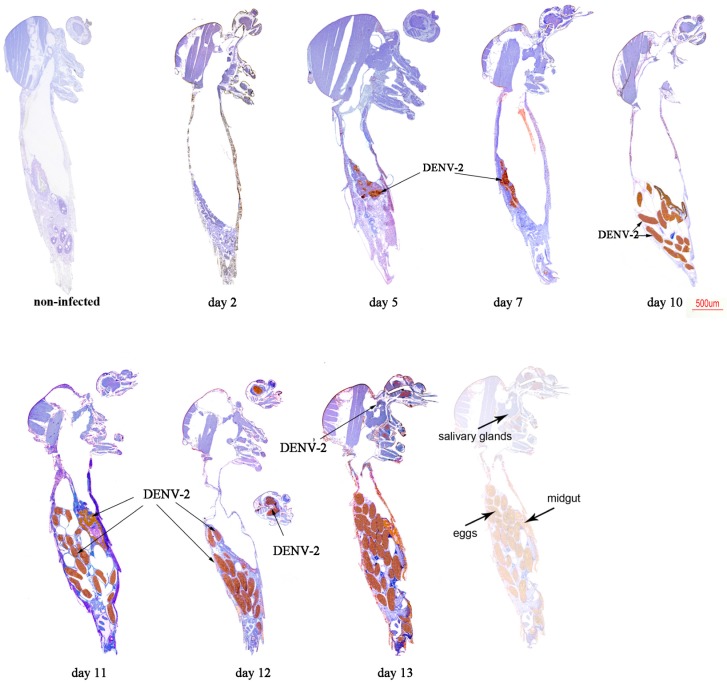
IHC staining of DENV-2 in paraffin-embedded mosquitoes at various times post-virus exposure. DENV-2 antigen stains brownish-red. A non-infected mosquito was used as control to determine non-specific staining using 4E11 anti-DENV-2 monoclonal antibody.

In DENV-2 infected mosquitoes, MG was found infected from day 5 post-exposure (Figure 5). While small foci of antigen were detected in the midgut epithelium at early time points, larger portions of adjacent antigen-positive epithelial cells were observed at later time points. Small foci of DENV-2 antigen were detected in SG only at day 13 post-exposure. Heads were found infected at day 12 post-virus exposure, whereas DENV-2 positive eggs were observed from day 10 to day 13 post-virus exposure.

**Figure 6:**
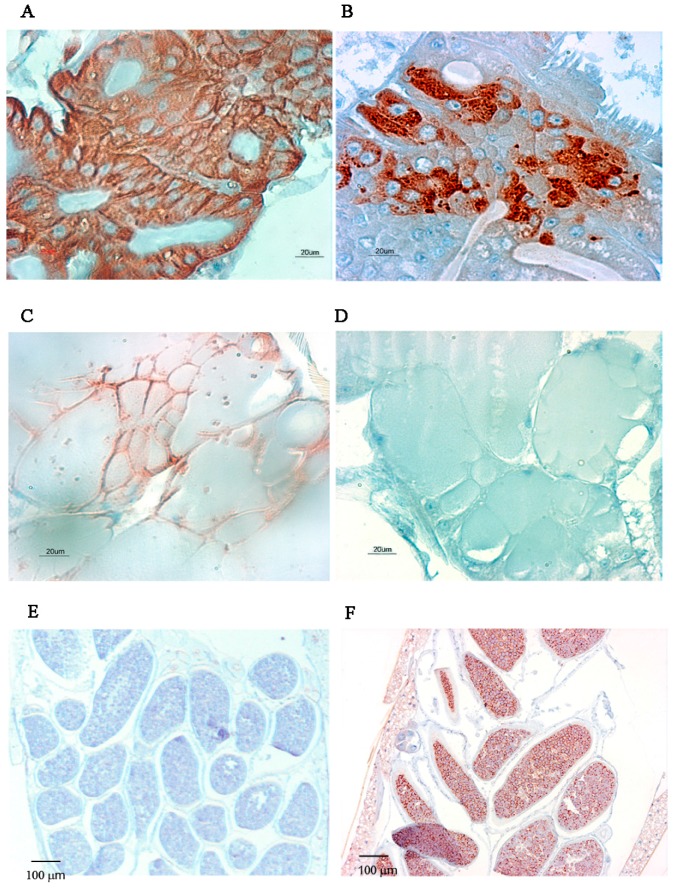
Distribution of CHIKV and DENV-2 antigens in mono-infected MG, SG and eggs by IHC at 7 dpve. (**A**,**B**) MG of CHIKV and DENV-2 infected mosquitoes, respectively, Scale bar = 20 µm; (**C**,**D**) SG of CHIKV and DENV-2 infected mosquitoes, respectively, Scale bar = 20 µm; (**E**,**F**) Eggs of CHIKV and DENV-2 infected mosquitoes, Scale bar = 100 µm. Antigens are stained brown.

We observed MG, SG and eggs of infected mosquitoes at higher magnification. For mono-infections, almost all MG epithelial cells were infected by CHIKV, whereas a few patches remained uninfected by DENV-2 at day 7 post-virus exposure (Figure 6). SG were infected by CHIKV as shown by the brownish-red staining observed in Figure 6, whereas we observed no staining of SG from DENV-2 infected mosquitoes, which is consistent with what was observed by indirect fluorescent antibody (IFA). Only eggs from DENV-2 orally exposed mosquitoes were stained whereas eggs were negative in CHIKV-exposed mosquitoes at 10 dpve.

**Figure 7:**
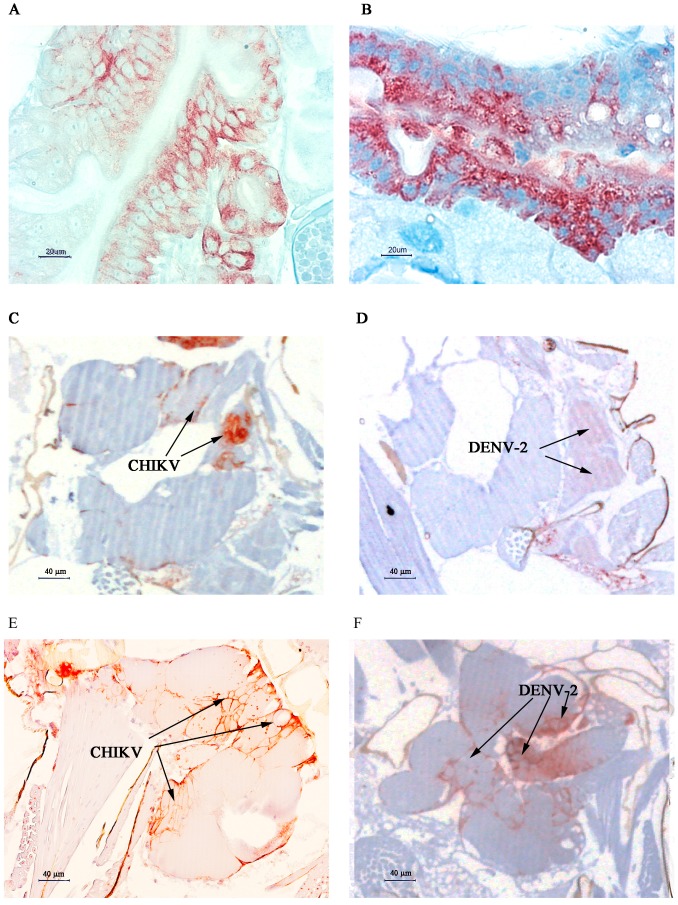
Distribution of CHIKV and DENV-2 antigens in the MG and SG of co-infected mosquitoes at 7 and 10 days post-virus exposure. Serial sections of the same organs were stained against CHIKV and DENV-2 antigens. (**A**,**B**) CHIKV and DENV-2 infected MG, respectively at 7 dpve, Scale bar = 20 µm; (**C**,**D**) CHIKV and DENV-2 infected SG respectively at 7 dpve, Scale bar = 40 µm; (**E**,**F**) CHIKV and DENV-2 infected salivary glands at 10 dpve, Scale bar = 40 µm. Antigens are stained in brownish-red.

When mosquitoes are co-infected by both viruses, a more intense labelling is observed at 7 dpve for DENV-2 antigen in the midgut whereas CHIKV labelling seems similar to mono-infected mosquitoes. CHIKV labelling is similar in the salivary glands, whereas a faint staining can be observed in one salivary lobe for DENV-2 at the same day compared to mono-infected DENV-2 salivary glands, for which no staining was observed. (Figure 7C,D). We observed an intense staining in both CHIKV and DENV-2 infected SG at 10 dpve.

### 2.3. Quantitative Reverse Transcription PCR (RT-qPCR) Analysis of CHIKV, DENV-2 RNA Levels of Mono- and Co-Infected Mosquitoes

We used RT-qPCR to measure the RNA copy number of each virus at various dpve in the MG and SG of mosquitoes infected with each virus separately or following co-infection with both. For co-infected mosquitoes, each isolated MG and SG was tested for the presence of the RNA of both viruses using specific RT-qPCR probes. 

**Figure 8:**
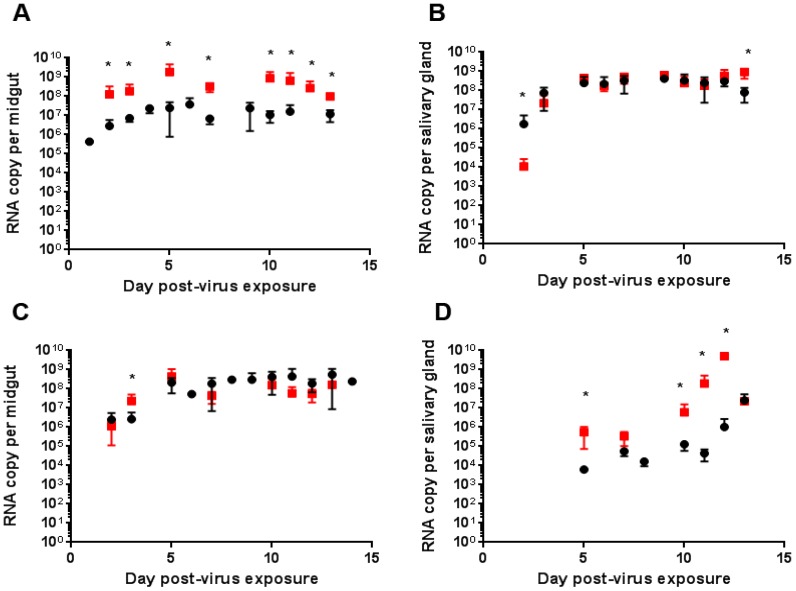
Replication kinetics of CHIKV and DENV-2 in mono or dually infected MG and SG. Viral RNA copy number was determined by RT-qPCR at the indicated times post-virus exposure. (**A**,**B**) CHIKV RNA was determined in MG (**A**) and SG (**B**) of mosquitoes mono-infected by CHIKV (●) or co-infected by CHIKV and DENV-2 (■), respectively); (**C**,**D**) DENV-2 RNA was determined in MG (**A**) and SG (**B**) of mosquitoes mono-infected by DENV-2 (●) or co-infected by DENV-2 and CHIKV (■). Eight mosquitoes were dissected at each time point and three replicates were performed. * indicates statistical differences between mon-infected and co-infected organs.

We first analysed the viral RNA copy number in mono-infected mosquitoes. RNA copy levels in the MG of mosquitoes infected by DENV-2 at days 2 and 3 post-virus exposure were more heterogeneous than those in CHIKV-infected MG (Figure 8). Indeed, we observed a significantly higher RNA copy number for CHIKV than DENV-2 at 3 dpve (*p* = 0.002). The CHIKV and DENV-2 RNA copy numbers in the MG of infected mosquitoes were similar at 7 dpve and remained constant for each virus until the end of the experiment. When mosquitoes were co-infected by CHIKV and DENV-2, all MG tested were positive for both viruses at all times post-infection. The replication kinetics of CHIKV were higher at all times tested when DENV-2 was present (*p* < 0.001), whereas the presence of CHIKV in the MG triggered an increase in DENV-2 RNA copy number at only day 3 post-virus exposure (*p* < 0.01) (Figure 8).

We also compared the replication kinetics for each virus in SG from mosquitoes infected with each virus separately or after co-infection (Figure 8). CHIKV replication was higher in the SG of CHIKV-infected mosquitoes than in those of co-infected mosquitoes at day 2 post-virus exposure, whereas it was higher in the SG of co-infected than singly infected mosquitoes at 13 dpve (*p* < 0.01). DENV-2 replication was higher in SG of co-infected than singly infected mosquitoes at 5, 10, 11 and 12 dpve (*p* < 0.01). 

## 3. Discussion

Mixed viral infections are ubiquitous in natural populations and may have significant but unpredictable biological and epidemiological consequences. These infections may be acquired simultaneously (co-infection) or result from two single infections at different times (superinfection). The first concurrent isolation of CHIKV and DENV-2 from a single blood specimen, taken from a patient in the acute phase of a dengue-like illness, occurred in Southern India in 1967 [27]. Since then, several cases of CHIKV/DENV co-infection have been reported in the literature. Two cases of imported infection in patients who had returned to Taiwan from Singapore were reported in 2010: one patient was co-infected with CHIKV and DENV-2 [22]. Co-infection with DENV and CHIKV was described in a child in 2012 [23]. A case of DENV-4 and CHIKV co-infection was also identified in a traveler returning from Luanda, Angola, in 2014 [24]. Recently, concurrent infections of Mayaro virus and DENV-4 were identified in Amazonia [28]. The small number of diagnosed co-infections makes it difficult to assess the severity of illness in patients with DENV/CHIKV co-infections. Additional clinical information is required to determine the influence of co-infections on the clinical expression of dengue and chikungunya fever. 

It is also important to understand the effect of co-infection on the vectors. Co-infections have been recorded in countries where several arboviruses circulate. For CHIKV and DENV, viremia in humans is sufficiently high to infect mosquitoes. Mosquitoes can be sequentially infected by biting CHIKV- and then DENV-infected patients or they can feed on co-infected patients. An *Ae. albopictus* specimen was found to be positive for both CHIKV and DENV-2 in the vicinity of co-infected patients, suggesting that co-infected mosquitoes may be competent vectors for transmitting both viruses. Recently, Rückert et al. determined the impact of simultaneaous exposure to arboviruses on infection and transmission by *Ae. aegypti* mosquitoes. Their results show a mild impact of co-infection on infection, dissemination and transmission rates [29]. In our study, we analyzed the effect of co-infection on the barriers of infection (midguts) and transmission (salivary glands) and were able to determine that co-infection was able to modify RNA replication in both barriers. 

Our study compared mono and co-infections of *Ae. aegypti* mosquitoes by CHIKV and DENV-2. We detected CHIKV and DENV-2 antigens by immunofluorescent imaging as early as day 3 post-virus exposure in MG from mono-infected mosquitoes. In SG, we detected CHIKV antigen as early as 4 dpve whereas DENV-2 staining was not detected before 10 dpve. The replication kinetics of DENV-2 and CHIKV were similar in MG of mono-infected mosquitoes, although we observed a lower RNA copy number at 3 dpve in DENV-2 infected MG. This may explain why a few patches of MG cells remained uninfected by DENV-2 at 7 dpve, as measured by IHC, whereas all cells were infected by CHIKV at the same time point. CHIKV replication in SG reached a plateau at 3 dpve in mono-infected mosquitoes, whereas we did not observe high levels of DENV-2 RNA before 11 dpve, in good agreement with our IFA and IHC results. Interestingly, immunostaining of sections of paraffin-embeded mosquitoes showed that DENV-2 antigen was detected in egg sections of several females over time post-viral exposure. This is quite surprising since vertical transmission of flaviviruses is known to happen in the fully formed egg during oviposition [30,31]. Our findings may suggest that infection with this particular strain of DENV-2 may infect the germinal tissues of the female mosquito (transovarial transmission) as described for other viruses as insect-specific flavivirus [32]. 

Co-infection of *Ae. aegypti* with CHIKV and DENV-2 led to a mixed infection for 100% of the mosquitoes studied, showing identical infection rates for singly and co-infected mosquitoes, and to facilitation of replication for both viruses. This was particularly true in the MG where the presence of DENV-2 increased CHIKV replication at all times post-infection. We observed the same effect for DENV-2, although only at day 3 post-virus exposure. In SG, we only observed increased CHIKV replication in the presence of DENV-2 at 13 dpve, whereas we observed the opposite at earlier time points post-infection. The presence of CHIKV increased DENV-2 replication at days 5, 10, 11 and 12 post-virus exposure, times at which the virus can be transmitted by the vector (end of the extrinsic incubation period). In agreement with these observations, we detected faint labelling of DENV-2 in SG collected at 7 dpve by IHC only when they were co-infected with CHIKV, suggesting a shorter time of infection of SG by DENV-2 in presence of CHIKV. We have confirmed this observation by another experiment in which *Ae. albopictus* mosquitoes were co-infected by CHIKV and DENV-4. Again, we observed a shorter time of infection of SG for DENV-4 in the presence of CHIKV (unpublished data). Our results demonstrate the facilitation of CHIKV and DENV-2 infection of the MG and SG of *Ae. aegypti* when the mosquitoes are co-infected.

Several studies have been performed in insect cells to assess the effect of co-infection with arboviruses belonging to distinct families on the outcome of viral replication. Competitive suppression of CHIKV replication in C6/36 cells during co-infection was shown to occur only when DENV is inoculated at high titres [33], which was not the case in our study. Mixed infections in C6/36 cells have no effect on La Crosse virus replication, whereas coinfection enhances Sindbis virus replication [34]. Mixed infections suppress the replication of both viruses in baby hamster kidney (BHK) cells, emphasizing the potential for mixed viral infections to modify arbovirus transmission and pathogenesis. Sindbis virus (SINV) blocks replication of DENV-4 in C6/36 *Ae. albopictus* cells with greater inhibition occurring when the two arboviruses are inoculated simultaneously than sequentially. In addition, studies have been performed in *Ae. albopictus* mosquitoes that were simultaneously exposed to both arboviruses. They had significantly lower DENV-4 infection and population dissemination rates than those exposed to DENV-4 alone [35]. These latter results contrast with those of our study. However, one parameter was different relative to our study. The CHIKV infection rate in our experimental design was very high, allowing a 100% infection rate, whereas the SINV infection rates were very low, ranging from 0–8% for the SINV only infections and 0−4% for the co-infections. This may explain, in part, the difference observed between the two studies.

RNAi and several conserved innate immune pathways act against arbovirus infection in mosquitoes. Mosquitoes possess specific antiviral strategies in the MG, hemolymph, SG, and neural tissues to control arboviral propagation. A recent study has reported differential protein modulation in the MG of *Ae. aegypti* infected with CHIKV and DENV-2 [36]. DENV-2 induced the modulation of more midgut proteins than CHIKV. Both viruses induced overexpression of proteins involved in cell protection, perhaps enhancing survival during infection. They also modulated the expression of other proteins, such as transferrin (CHIKV and DENV-2), hsp60 and alpha glucosidase (DENV-2), which may favor viral survival and replication inside the MG. These results suggest that the viruses may act in synergy to influence MG metabolism.

## 4. Materials and Methods

### 4.1. Mosquitoes

*Aedes aegypti* (Liverpool strain) were maintained at 28 ± 1 °C, at a relative humidity of 80%, with a light/dark cycle of 16 h/8 h. Larvae were reared in pans containing cat food (beef and Chicken) in 1 L of tap water. Adults were provided with a 10% sucrose solution *ad libitum*.

### 4.2. Viruses

CHIKV 06.21, isolated in November 2005 from a new-born male from La Reunion presenting symptoms of meningo-encephalitis [37], was used for all experiments. This strain contains the amino-acid change A→V at position 226 of the E1 glycoprotein (E1-226V). The titre of the frozen stock virus was estimated to be 10^9^ plaque-forming units (PFU)/mL. The DENV-2 strain, provided by Leon Rosen (Institut Pasteur, Paris, France) was isolated in 1974 from human serum in Bangkok (Bangkok, Thailand) (D2BN32). Viral stocks were produced by inoculating *Ae. albopictus* cells (clone C6/36) with triturated infected mosquitoes. Titration of the viral stock was carried out in *Ae. aegypti* (Paea strain) by inoculating serial dilutions of the supernatant intra-thoracically. Mosquito infection was detected by an indirect fluorescent antibody (IFA) assay on head squashes. Titres were calculated by the 50% endpoint method and expressed as the 50% mosquito infectious dose (MID 50/mL). The titre of the stock virus was estimated to be 10^9.5^ PFU/mL.

### 4.3. Oral Infections of Mosquitoes and Dissections

Seven day-old female mosquitoes were deprived of sucrose 24 h prior to the infectious blood meal. They were then allowed to feed for 15 min through a chicken skin membrane covering glass feeders maintained at 37 °C. The infectious blood meal was comprised of two thirds washed rabbit erythrocytes, one third viral suspension, and adenosine triphosphate (ATP) (as a phagostimulant) at a final concentration of 5 × 10^−3^ M. The infectious blood had a titre of 10^7.5^ PFU/mL (CHIKV 06.21) or 10^9^ MID_50_/mL (DENV-2). Infections were repeated three times and at least 8 mosquitoes were dissected at each time point.

### 4.4. Reverse Transcription Quantitative PCR (RT-q PCR)

Total RNA from mosquitoes or MG was extracted using the Nucleospin® RNA II kit (Macherey-Nagel, Bethlehem, PA, USA) according to the manufacturer’s instructions. RNA was eluted in 40 μL RNAse-free H_2_O by centrifugation at 11,000× *g* for 1 min.

Synthetic RNA transcripts for CHIKV and DENV-2 were generated to construct a standard curve. The targeted regions in the CHIKV and DENV-2 sequences were amplified by PCR and the product ligated into the pCR II TOPO vector (Invitrogen, Carlsbad, CA, USA). The resulting plasmid was linearized using EcoRI restriction enzyme and purified using the QIAquick PCR purification kit. RNA transcripts were prepared in vitro using RiboMAX™ Large Scale RNA Production Systems (Promega, Madison, WI, USA) appropriate for either SP6 or T7 RNA polymerase. Residual DNA was eliminated by several DNAse treatments (Turbo DNA-free, Ambion, Foster City, CA, USA). After spectrophotometric quantification, the RNA transcript solution was stored at −80 °C.

One-step reverse transcription quantitative RT-q PCR (RT-qPCR) was performed using the Power Sybr Green RNA-to-Ct one step kit (Applied Biosystems, Carlsbad, CA, USA). The CHIKV primers were selected in the E2 structural protein regions: sense Chik/E2/9018/+ (CACCGCCGCAACTACCG); anti-sense Chik/E2/9235/− (GATTGGTGACCGCGGCA). The DENV-2 primers were selected as described in Lanciotti et al. [38]: sense (CGCCACAAGGGCCATGAACAG), antisense (TCAATATGCTGAAACGCGCGAGAAACCG).

RT-qPCR was performed using a Fast Real-Time PCR Systems 7500 with 7500 v.2.0.1 software (Applied Biosystems). The thermal cycling conditions were as follows: a reverse transcription step at 48 °C for 30 min, an inactivation step of the RT/RNAse enzyme at 95 °C for 10 min, followed by 40 cycles of 95 °C for 15 s, 60 °C for 1 min, a final denaturation step where the temperature increased from 60 to 95 °C during 20 min, and a step of 15 s at 95 °C. Signals were normalized to the standard curve using serial dilutions of synthetic RNA transcripts for CHIKV and DENV-2. Normalized data were used to estimate the transcript copy number in infected mosquito organs.

### 4.5. Immunofluorescence

After dissection in PBS, MG and SG were placed on a slide. PBS was removed and MG were fixed in acetone for 15 min, dried, and stored at 4 °C until use. SG were fixed with 4% paraformaldehyde for 15 min, rinsed with PBS, dried, and stored at 4 °C until use. For indirect IFA experiments, MG and SG were rehydrated in PBS for 3 × 5 min, and then incubated for 15 min (SG) or 1 h (MG) in Triton X-100 (0.2%). They were washed again with PBS (3 min × 5 min) and incubated for 30 min in PBS containing 5% BSA. The slides were drained and incubated overnight at 4°C with anti-DENV-2 protein E 3H5 diluted 1:400 in PBS, or Cy3 conjugated anti-chikungunya E 3E4 protein diluted 1:500 in PBS, and then washed with PBS (3 min × 5 min) with shaking. DENV-2 infected organs were incubated for 1 h with 1Alexafluor 488 goat anti/mouse (Invitrogen) diluted 1:500 in PBS, and washed with PBS. For co-infections, MG and SG were first incubated with anti-DENV-2 protein E 3H5, and then Alexafluor 488 goat anti/mouse antibody. After washing, mosquito organs were then incubated with Cy3 conjugated anti-chikungunya E 3E4 protein. The actin network was stained with Alexafluor 633 phalloidin (Invitrogen) (diluted 1:40 in PBS) for DENV-2 staining or Alexafluor 488 phalloidin for CHIKV staining. In case of co-infection, actin network was stained with Alexafluor 633 phalloidin. After washing, a drop of Prolong gold antifade (Invitrogen) was applied on each slide and a coverslide was placed on top. All preparations were examined by confocal microscopy (Zeiss LSM 510 Meta and TCS SP5 Leica Microsystems (Wetzlar, Germany). 

### 4.6. Histological Observations

At each indicated time post-virus exposure, 5 infected and control females were killed and fixed in Carnoy solution (3 vol. chloroform, 1 vol. absolute ethanol, 1 vol. acetic acid). Samples were then dehydrated as follows: 8 h in absolute ethanol, 17 h in solution 1 (55% n-butanol/40.5% absolute ethanol in H_2_O), 8 h in solution 2 (75% n-butanol/22.5% absolute ethanol in H2O) and finally 2–3 days in n-butanol. Mosquitoes were embedded in paraffin. Sections (5 µm) were stained with hematoxylin and eosin, periodic acid Schiff, and Gordon sweet stains according to [39]. Immunohistochemical analysis was performed by using an anti-CHIKV or DENV-2 polyclonal mouse ascitic fluid at a dilution 1:750. Briefly, tissue sections were immersed in 200 mL of citrate and incubated three times for 5 min in a microwave at 650 W before staining. The streptavidin peroxydase method with AEC (amino ethyl carbozole) as a chromogen was used to detect the secondary antibody (Envision system labeled Polymer-HRP antimouse, Agilent, Santa Clara, CA, USA). Slides were observed with light microscopy.

### 4.7. Statistical Analyses

Simstat software was used for all analyses that were performed using the Mann-Whitney non-parametric test.

## 5. Conclusions

We have shown that mixed CHIKV and DENV infection facilitates viral replication in *Ae. aegypti*. These findings are important, as areas of co-circulation of various arboviruses are expanding throughout the world. An increase in such co-infections may alter the infectivity or pathogenicity of both viruses. The outcome of these mixed infections must be further studied to increase our understanding of pathogen-pathogen interaction in the host cell. These results also pave the way to understanding the antiviral response when more than one arbovirus is present in the mosquito.

## Abbreviations

MG	Midgut
SG	Salivary gland
IFA	Immunofluorescence assay
CHIKV	Chikungunya virus
DENV-2	Dengue virus serotype 2
IHC	Immunohistochemistry
dpve	Day post-virus exposure
